# Identification and characterisation of *de novo* germline structural variants in two commercial pig lines using trio-based whole genome sequencing

**DOI:** 10.1186/s12864-023-09296-3

**Published:** 2023-04-18

**Authors:** Marije J. Steensma, Y. L. Lee, A. C. Bouwman, C. Pita Barros, M. F.L. Derks, M. C.A.M. Bink, B. Harlizius, A. E. Huisman, R. P.M.A. Crooijmans, M. A.M. Groenen, H. A. Mulder, C. M. Rochus

**Affiliations:** 1 Wageningen University & Research Animal Breeding and Genomics, P.O. Box 338, Wageningen, 6700 AH the Netherlands; 2 Topigs Norsvin Research Center, Schoenaker 6, Beuningen, 6641 SZ the Netherlands; 3 Hendrix Genetics, P.O. Box 114, Boxmeer, 5830 AC the Netherlands; 4 University of Guelph, Centre for Genetic Improvement of Livestock, 50 Stone Rd E, Guelph, O N N1G 2W1 Canada

**Keywords:** *De novo* mutation, Structural variation, Germline mutation, *Sus scrofa*, Trios, Whole genome sequencing, Three-generational, Genomics

## Abstract

**Background:**

*De novo* mutations arising in the germline are a source of genetic variation and their discovery broadens our understanding of genetic disorders and evolutionary patterns. Although the number of *de novo* single nucleotide variants (dnSNVs) has been studied in a number of species, relatively little is known about the occurrence of *de novo* structural variants (dnSVs). In this study, we investigated 37 deeply sequenced pig trios from two commercial lines to identify dnSVs present in the offspring. The identified dnSVs were characterised by identifying their parent of origin, their functional annotations and characterizing sequence homology at the breakpoints.

**Results:**

We identified four swine germline dnSVs, all located in intronic regions of protein-coding genes. Our conservative, first estimate of the swine germline dnSV rate is 0.108 (95% CI 0.038–0.255) per generation (one dnSV per nine offspring), detected using short-read sequencing. Two detected dnSVs are clusters of mutations. Mutation cluster 1 contains a *de novo* duplication, a dnSNV and a *de novo* deletion. Mutation cluster 2 contains a *de novo* deletion and three *de novo* duplications, of which one is inverted. Mutation cluster 2 is 25 kb in size, whereas mutation cluster 1 (197 bp) and the other two individual dnSVs (64 and 573 bp) are smaller. Only mutation cluster 2 could be phased and is located on the paternal haplotype. Mutation cluster 2 originates from both micro-homology as well as non-homology mutation mechanisms, where mutation cluster 1 and the other two dnSVs are caused by mutation mechanisms lacking sequence homology. The 64 bp deletion and mutation cluster 1 were validated through PCR. Lastly, the 64 bp deletion and the 573 bp duplication were validated in sequenced offspring of probands with three generations of sequence data.

**Conclusions:**

Our estimate of 0.108 dnSVs per generation in the swine germline is conservative, due to our small sample size and restricted possibilities of dnSV detection from short-read sequencing. The current study highlights the complexity of dnSVs and shows the potential of breeding programs for pigs and livestock species in general, to provide a suitable population structure for identification and characterisation of dnSVs.

**Supplementary Information:**

The online version contains supplementary material available at 10.1186/s12864-023-09296-3.

## Background

*De novo* mutations (DNMs) are spontaneous mutations in the germline and a source of genetic variation that occur during gametogenesis [1]. The new alterations in the genome are genetic variants absent in the somatic cells of the parents and present in the germline of their offspring. Hereafter offspring where DNMs are detected, are referred to as proband [2]. Depending on the method used for DNM detection, mutations arising in the early fertilized egg cell during embryogenesis, resulting in an individual carrying the mutation in some, but not all tissues and organs, are often mistaken as germline DNM [3, 4]. This phenomenon is known as mosaicism, and depending on when mutations have occurred, mosaicism may be restricted to soma, germline or involving both [5]. When mutations occur in the germline, as in DNMs, they can be transmitted to the next generation. The genetic variation introduced by the mutation allows for continued selection and adaptation in a population, with the tradeoff that DNMs can be deleterious and impact the fitness of an individual [6]. Therefore, identifying DNMs can broaden the understanding of genetic disorders and evolutionary patterns in the short and long term [7].

The origins and patterns of *de novo* single nucleotide variants (dnSNVs) have been studied in humans and other primates [8–10]. In humans, the germline dnSNV rate is estimated at approximately 1 × 10^− 8^ per site per generation, giving rise to 44 to 82 dnSNVs per offspring [4, 11–13]. Several mechanisms are known to cause dnSNVs, mostly involving DNA replication [14]. The male germline undergoes continuous germ cell divisions from puberty onwards, whereas the female germline does not [9]. This has likely resulted in the paternal bias in the origin of dnSNVs that has been found in humans and chimpanzees [9, 11, 15]. Moreover, approximately 2 to 3% of all dnSNVs in offspring were found to occur together (< 20 kb) as clustered mutations [8, 16, 17]. These clustered dnSNVs are equally abundant on maternal and paternal gametes and have mutation spectra distinct from non-clustered DNMs, suggesting different underlying mechanisms [8, 18, 19]. Furthermore, studies have revealed dnSNV rates in several species, including birds, cattle, fish and wolves [2, 20–22].

*De novo* structural variants (dnSVs) are predicted to occur a hundred-fold less frequently than dnSNVs [6]. Structural variants (SVs) are altered DNA segments larger than 50 base pairs (bp), that are a change in copy number (deletion, duplication, insertion), chromosomal location (translocation) or orientation (inversion) [23]. SVs can introduce new changes in gene dosage and structure, and affect gene expression and function by gains or losses of DNA segments [24, 25]. Recent studies have shown that dnSVs are associated with rare genetic disorders in humans, including autism and schizophrenia [3, 6, 26]. Additionally, SVs have been suggested to contribute to the phenotypic variation of economically important traits in livestock species [27–29], emphasizing the importance of detecting dnSVs. However, dnSV rates have only been reported in humans [6] and rhesus macaques [7], with a large variance in estimates of dnSV rates in humans [6]. Contrary to dnSNV detection, the identification of dnSVs in populations remains a major challenge. The detection of SVs is restricted by limited sensitivity of micro-array and sequencing-based approaches [30], which has resulted in a limited understanding of the dnSV rate.

The population structure of livestock species is helpful to study DNMs. In contrast to humans, most livestock species, including the domestic pig, have large family sizes and relatively short generation intervals. In addition, their phenotypes, genotypes and relationships are routinely collected in breeding programs, making it possible to investigate the impact of DNMs on the phenotypes of the offspring. While mutation rates in livestock species have not been widely studied, such studies could contribute to our understanding of genetic disorders and evolutionary patterns.

The aim of our study was to detect and characterize dnSVs in two commercial pig lines using trio-based whole genome sequencing (WGS) data. We analysed the rate of DNMs for three major classes of SVs: deletions, duplications and inversions. We provide a first, conservative estimate of swine germline dnSV rate in pigs using short-read WGS data. We also provide fine-scale molecular characterisation of these identified dnSVs, including identification of their parent of origin, functional annotations and characterisation of their sequence homology at the breakpoints.

## Results

### Candidate dnSVs

A total of 90,031 autosomal SVs, including 46,478 deletions, 6,541 duplications, 5,977 inversions and 31,035 breakend class variants were identified. We found significant positive correlations between the mean sequencing depth and number of duplications (r = 0.620, P < 0.0001), inversions (r = 0.405, P < 0.01) and break end class variants (r = 0.814, P < 0.0001) in line 1, and a significant positive correlation between the mean sequencing depth and the number of breakend class variants (r = 0.593, P < 0.0001) in line 2. (Additional file 1: Figure S1). Additionally, a few samples show an excess of candidate inversions compared to the other samples (Additional file 1: Figure S1). Further analyses focused on deletions, duplications and inversions. Structural variants in the breakend class were not considered because this class contains non-canonical types of SVs which are difficult to map and interpret. After filtering for genotype-based Mendelian inconsistencies, a total of 1,521 deletions, 359 duplications, and 4,082 inversions remained. Six probands had an excess call of candidate *de* novo inversions and all had DNA extracted from semen. A majority of these *de novo* inversions overlap with repeats and were similarly called in multiple probands with an excess of *de novo* inversions (semen samples). Identical dnSVs found in multiple probands are likely false positives, because dnSVs are unique and expected to be a one-time event. In subsequent filtering steps we removed spurious sites based on allele count filters, changes in read-depth, number of reads and identical candidate dnSVs found in multiple probands (see materials and methods). After these filters, we retained 163 candidate dnSVs, including 67 deletions, 15 duplications and 81 inversions in 37 trios that were manually inspected using Integrative Genomics Viewer (IGV) [31].

### Identified dnSVs

**Fig. 1:**
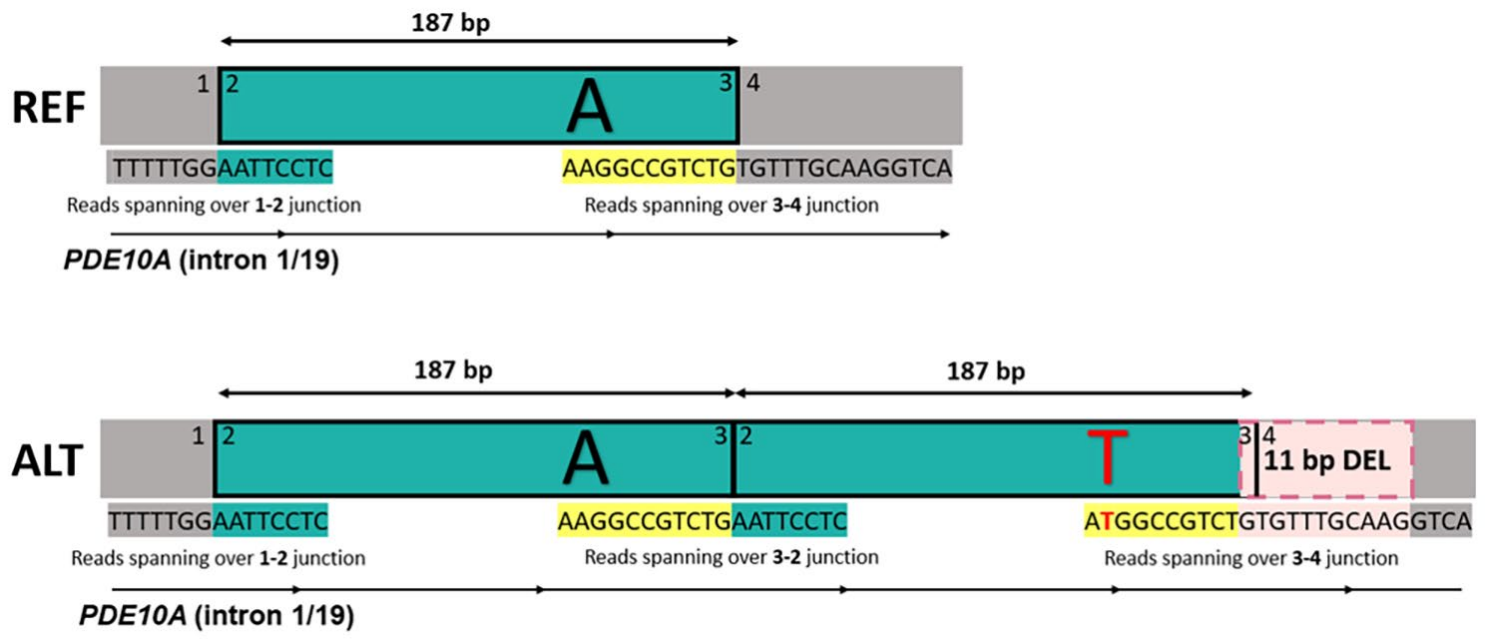
**Reconstruction of mutation cluster 1.** REF represents the reference allele. ALT represents the alternate allele involving a 187 bp *de novo* duplication, a *de novo* single nucleotide variant (dnSNV) (A > T) and an 11 bp *de novo* deletion. Reads are shown which are spanning over different junctions (1–4). Intron 1 of the gene *PDE10A* is shown, which overlaps with this region. IGV screenshot of mutation cluster 1 can be found in Additional file 1: Figure S2

**Fig. 2:**
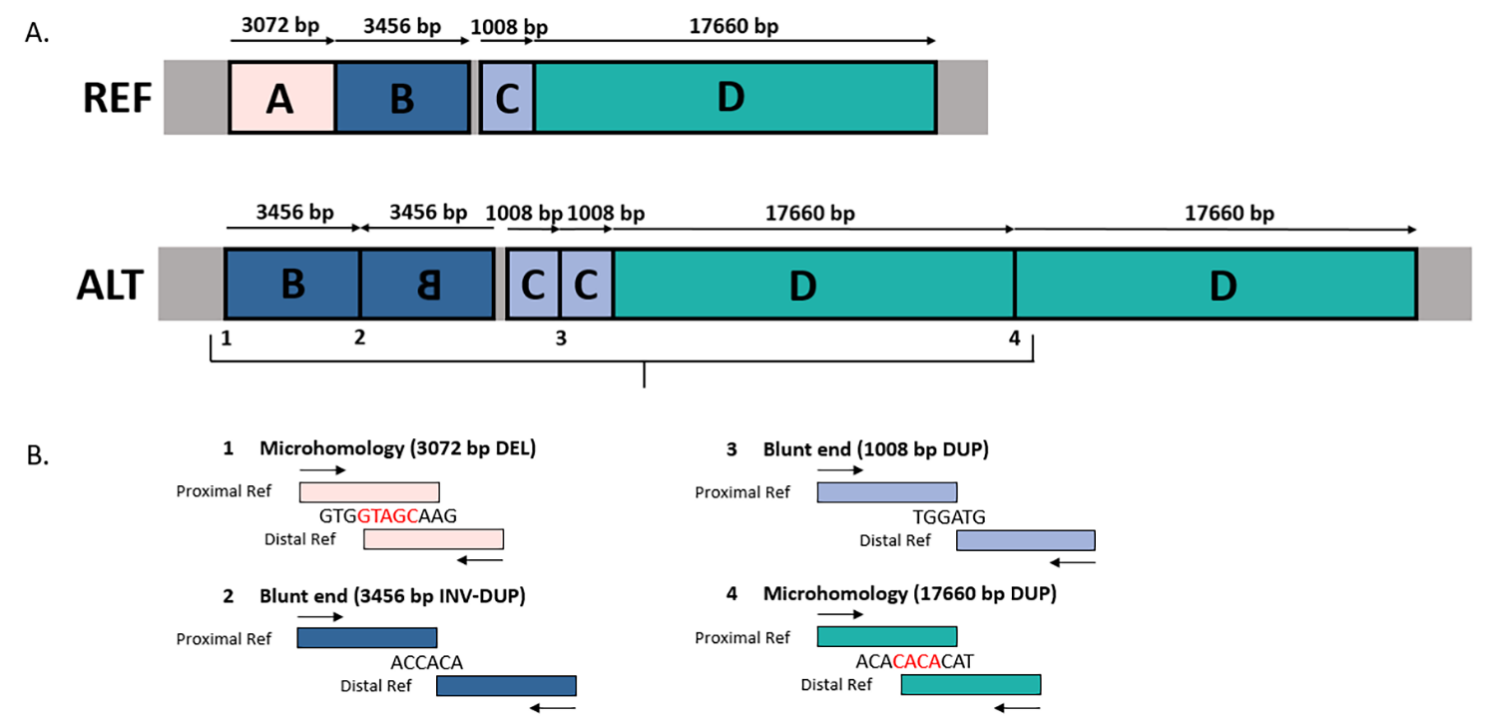
**Reconstruction of mutation cluster 2. A.** The reference genome (REF) compared to the alternate mutation cluster 2 (ALT). Mutation cluster 2 represents the structure: Deletion (A) – Inverted duplication (B) – Normal (196 bp) – Duplication (C) – Duplication (D). The 610 bp overlap of deletion A and duplication B and the 42 bp overlap of duplication C and D are not presented in this figure for simplification. The figure is not to scale. **B.** Shows the presence of sequence homology (micro-homology) or no homology (blunt ends) at the junctions of each dnSV. Bases indicated in red represent homology. DEL = deletion, DUP = duplication, INV-DUP = inverted duplication. Integrative Genome Viewer screenshots of mutation cluster 2 can be found in Additional file 1: Figure S3

Based on manual inspection in IGV, we identified four high evidence germline dnSVs in 37 pig trios, ranging between 64 bp and 25 kb (Table 1). We estimated a swine germline dnSV rate of 0.108 (95% CI 0.038–0.255) per generation (one dnSV per nine offspring). Of the four detected dnSVs we identified, two are clustered mutations, consisting of multiple mutation events. Mutation cluster 1 is 197 bp in size and contains a 187 bp *de novo* duplication, a dnSNV within the duplication and an 11 bp *de novo* deletion at the distal breakpoint of the duplication (Fig. 1). Mutation cluster 2 consists of one *de novo* deletion and three *de novo* duplications, of which one duplication is inverted (Fig. 2A). Mutation cluster 2 is 25 kb in size, whereas the size of mutation cluster 1 (197 bp) and the other two detected dnSVs are smaller (64 and 573 bp). IGV screenshots of all identified dnSVs can be found in Additional file 1: Figures S2 to S5. Moreover, we detected a 276 bp mosaic deletion in the proband (Table 1). This mosaic deletion contains heterozygous SNPs showing a 2:1 ratio in the proband and a 1:1 ration in both parents, indicating that ~ 25% is deleted (Additional file 1: Figure S6). This deletion was determined as mosaic, because it deviates from the 50% deletion expected for true germline dnSVs, and is therefore not included in calculating the dnSV rate nor described further.

For the four dnSVs, we analysed their genic overlap. All identified dnSVs partially overlap with intronic regions of protein-coding genes (Ensembl release 107, July 2022 and NCBI release 106, May 2017) (Table 1): Mutation cluster 1 overlaps with phosphodiesterase 10 A (*PDE10A*), mutation cluster 2 overlaps with the solute carrier family member 2 (*SLC14A2*), the 573 bp duplication overlaps with the BRD4 (Bromodomain Containing 4) interacting chromatin remodeling complex associated protein like (*BICRAL*) and the 64 bp deletion overlaps with the NCK (non-catalytic region of tyrosine kinase adaptor) associated protein 5 (*NCKAP5*).

**Table 1:** Identified *de novo* structural variants (dnSVs) from whole genome sequencing (WGS) data of 37 trios

dnSV type	Position	Size (bp)	Parent of origin	Locus	Putative mechanism	PCR √^1^	Offspring √^2^
Mutation cluster 1		197	n.a.	*PDE10A*		Yes	n.a.
-Duplication	Chr1:2940197–2,940,384	187		(intron 1/19)^3^	NON-HOM		
-dnSNV	Chr1:2940375	1			n.a.		
-Deletion	Chr1:2940383–2,940,394	11			n.a.		
Mutation cluster 2		25,196	Paternal	*SLC14A2*		n.a.	n.a.
-Deletion	Chr1:95207720–95,210,792	3072		(intron 3/12)^4^	MICRO-HOM		
-Duplication	Chr1:95210182–95,213,638	3456			Unidentified		
-Duplication	Chr1:95213834–95,214,842	1008			NON-HOM		
-Duplication	Chr1:95214800–95,232,460	17,660			MICRO-HOM		
Duplication	Chr7:37837360–37,837,933	573	n.a.	*BICRAL* (intron 1/11)^3^	NON-HOM	No	Yes
Deletion	Chr15:18965177–18,965,241	64	n.a.	*NCKAP5* (intron 4/17)^4^	NON-HOM	Yes	Yes
Mosaic deletion	Chr11:53586793–53,587,069	276	n.a.	Intergenic	MICRO-HOM	No	n.a.

^1^dnSV validated by PCR.

^2^dnSV validated in one sequenced offspring of the proband.

^3^Source: Ensembl.

^4^Source: NCBI.

n.a.: not available. NON-HOM = non-homology. MICRO-HOM = micro-homology.

### Validated dnSVs

We searched for the detected dnSVs in the public Ensembl SV database (release 107, July 2022) [32] and a pig-specific variation database, PigVar [33], and found that all four detected dnSVs have not been publicly reported before.

Three out of the four detected dnSVs were validated. We designed PCR primers for three of the four detected dnSVs: (i) the 64 bp deletion, (ii) the 573 bp duplication, (iii) and the 187 bp duplication within mutation cluster 1 (Additional file 2: Table S1). Additionally, PCR primers were designed for the 276 bp mosaic deletion (Additional file 2: Table S1). The 187 bp duplication within mutation cluster 1 and the 64 bp deletion were validated to be present in the heterozygous state in the proband and some of its offspring through PCR (Table 1). The 573 bp duplication and the 276 bp mosaic deletion were not validated in proband and its offspring as PCR amplification showed no variation (Table 1, Additional file 2: Table S1).

It would be possible to validate the transmission of germline dnSVs if the proband had sequenced offspring available. The 64 bp deletion and the 573 bp duplication were found in probands where three generations of sequence data were available and the transmission of both dnSVs was validated in the sequenced offspring of the proband (Table 1). We were unable to validate mutation cluster 2 because it was too complex for PCR primer design and the proband did not have any sequenced offspring available (Table 1).

### Parent of origin

The presence of informative SNPs located within dnSVs can aid in identifying the parent of origin. Mutation cluster 2, consisting of one deletion and three duplications, has informative SNPs (Additional file 1: Figure S3). The informative SNPs within the deletion of mutation cluster 2 are homozygous for the alternate or reference allele in the proband, and homozygous for the other allele in the father. The informative SNPs within the duplications of mutation cluster 2 are heterozygous with a 2:1 ratio in the proband where the allele with more than expected number of reads (causing the 2:1 ratio) came from the father. Hence, we identified mutation cluster 2 as originating from the paternal gamete (Table 1). The other three identified germline dnSVs had no informative SNPs located within the breakpoints of the dnSVs and therefore the parent of origin could not be determined.

### Mutation mechanisms

We were able to categorize the dnSVs by the degree of sequence homology surrounding the breakpoints into two broad categories: non-homology (NON-HOM) (0 to 1 bp) and micro-homology (MICRO-HOM) (2 to 15 bp) (Table 1). Based on these definitions, the 187 bp duplication within mutation cluster 1, the 64 bp deletion, and the 573 bp duplication show no sequence homology at the breakpoints. The deletion and duplications within mutation cluster 2 shows different sequence homology at the breakpoints (Fig. 2B). A 5 bp and a 4 bp micro-homology was found at the breakpoints of the 3,072 bp deletion and 17,660 bp duplication, respectively. No sequence homology was found at the breakpoints of the 1,008 bp duplication and the 3,456 bp inverted duplication. The latter shows a unique feature which was too complex to interpret.

## Discussion

The current study focused on identification and characterisation of dnSVs. Here, we report a conservative, first estimate of the swine germline dnSV rate of 0.108 per generation (one dnSV per nine offspring) detected using short-read sequencing data. Our estimate is similar to the rate reported humans (0.122 per generation) [6]. We identified four germline dnSVs, in 37 sire-dam-proband trios, all of which overlapped with intronic regions of protein-coding genes. Two of the four germline dnSVs are clusters of *de novo* mutations. As in humans [30], clusters of mutations were also observed, where complex mutation clusters consisted of combinations of dnSVs or dnSNVs. We were able to validate two out of three tested dnSVs through PCR, the 64 bp deletion and mutation cluster 1. Additionally, the 64 bp deletion and the 573 bp duplication were found in probands where three generations of sequence data were available and the transmission of both dnSVs were confirmed in the sequenced offspring of the proband. We suggest future studies to use larger cohorts with three generations of long-read sequence data, as it will aid in more accurate detection and validation of germline dnSVs.

### Mutation clusters

Two of the four identified dnSVs co-occur with other DNMs and we describe them as mutation clusters. Mutation cluster 1 consists of a *de novo* duplication, a dnSNV and a *de novo* deletion. Mutation cluster 2 consists of one *de novo* deletion and three *de novo* duplications of which one is inverted. The observation of mutational clusters is consistent with a dnSV study in humans, which also found dnSVs consisting of clusters of deletions, duplications and inversions occurring as single events as well as dnSVs co-occurring with dnSNVs [30]. In humans, these mutation clusters could be explained by mutation hotspots, which are regions in the genome where there is an increased number of variants segregating in the population near the breakpoints of dnSVs [30]. A recent study in humans showed that mutation hotspots are enriched with unbalanced dnSVs [34]. Additionally, another study in humans showed that SNVs occurred during the formation of SVs and thereby are enriched in SV hotspots [35]. However, mutation hotspots are species-specific and for pigs yet unknown.

### Intronic dnSVs and gene functions

Copy number variations (CNVs; deletions, duplications, insertions) have been shown to capture 17.7% of the total variation in gene expression in humans [36]. The CNVs with large effect size on gene expression were mostly the ones disrupting coding sequence or impacting the regulatory landscape of the region where those CNVs occur [36]. In the current study, we found that all detected dnSVs are located within intronic regions (non-coding sequences) of protein-coding genes. Therefore, we should not necessarily expect these detected dnSVs to contribute to variation in gene expression. Nevertheless, introns encompass half of the non-coding genome and are known to contain important regulatory elements [37]. A study on intronic SVs (deletions, duplications, insertions) in humans found that intronic deletions can result in repressed or enhanced gene expression [37]. Thus, to interpret possible effects of dnSVs it is important to understand the genes with which the dnSVs overlap and their functions.

Previous studies reported functions and diseases associated with the genes overlapping with the dnSVs found in the current study. First, the 573 bp duplication overlaps with the BRD4 (Bromodomain Containing 4) interacting chromatin remodeling complex associated protein like (*BICRAL*), which is a component of the ATP-dependent SWI/SNF (SWItch/Sucrose Non-Fermentable) chromatin remodelling subcomplex GBAF (GLTSCR1 like containing BRG1/BRM-associated factor). This subcomplex performs important enzymatic activities leading to changes in chromosome structure by altering DNA-histone contacts within a nucleosome [38]. *BICRAL* is associated with Coffin-Siris Syndrome 3 in humans which causes congenital malformation and can cause feeding difficulties and poor growth [39]. *BICRAL* homozygous knockout mice show an embryonic lethal phenotype [40, 41]. Second, mutation cluster 1 overlaps with phosphodiesterase 10 A (*PDE10A*), which plays a role in signal transduction by regulating the intracellular concentration of cyclic nucleotides. This gene is associated with a hyperkinetic movement disorder in humans [42] and with striatal degeneration in humans [43]. *PDE10A* knockout mice exhibit a resistance to diet-induced obesity and multiple behaviour abnormalities, e.g., decrease in exploratory locomotor activity [41, 44, 45]. Mice homozygous for the *PDE10A* knockout are viable and fertile, although breeding with two homozygote *PDE10A* knockout mice results in reduced litter sizes [44, 45]. Third, the 64 bp deletion overlaps with the NCK (non-catalytic region of tyrosine kinase adaptor) associated protein 5 (*NCKAP5*). This protein-coding gene is uncharacterized but associated with attention deficit-hyperactivity disorder [46] and drug-induced lupus erythematosus in humans [47]. Last, mutation cluster 2 overlaps with the solute carrier family member 2 (*SLC14A2*). This gene is involved in the urea transport family [48] and associated with the following diseases: bone chondrosarcoma [49] and susceptibility to yaws, a primary bacterial infectious disease that infects the skin, bones, and joints [47]. The two genes, *BICRAL* and *PDE10A*, for which the effects in knockout mice have been investigated, only appear if the mice were homozygous for the knock-out allele. In the current study, the pigs carrying the dnSV are all heterozygous for the dnSV, and likely have normal phenotypes since they were selected as breeding candidates for the two commercial pig lines. Nevertheless, homozygous offspring can occur in subsequent generations and might show a more visible effect on the phenotype.

### Mutation mechanisms

We found that three out of four detected dnSVs are mediated by mechanisms that do not require sequence homology at the breakpoints. This agrees with a study in humans, which showed that 75% of the detected dnSVs lacked sequence homology at the breakpoints [6]. We found that mutation cluster 2 likely arises through several mechanisms, including non-homology, micro-homology based mechanisms and an unidentified complex mechanism. Other studies have shown that micro-homology at the breakpoints, including micro-homology-mediated break-induced replication, can cause various complex rearrangements [50, 51]. In some other SV studies, classifications of the underlying forces driving dnSVs are more specific [30, 52]. These studies identify classes like non-homologous end joining, micro-homology break-induced replication, mobile element insertion and non-allelic homologous recombination [30, 52]. Because identifying the exact causal mechanisms is complex and mechanisms can act together [53], we only characterized the detected dnSVs based on sequence homology at the breakpoints and grouped them in a non-homology or micro-homology category. We were not able to find macro homology, like non-allelic homologous recombination, at the breakpoints as this is difficult to detect with short-read sequencing data [6]. Thus, to get a better understanding of the underlying mechanisms driving the evolution of dnSVs, a more comprehensive analysis using long-read sequencing approaches is required [54].

### Limitations to detect dnSVs with short reads

There are several challenges associated with detecting dnSVs using short-read sequencing, which likely resulted in undetected dnSVs (false negative) and false positive variant calls [55, 56]. In this study, we saw a high rate of false positive variant calls (97.5%) as only four out of the 163 candidate dnSVs (2.5%) were identified as high evidence germline dnSVs. This is mainly due to the excessive number of candidate (*de novo*) inversions in a few samples (Additional file 1: Figure S1), of which the majority overlaps with repeated regions. It is difficult to map short-reads uniquely to repetitive regions [57, 58] and paired-end short-read alignments are not always able to reveal the complete structure of SVs [56]. One approach to overcome these short-read deficiencies, and thereby also lower the rate of false positive and false negative variant calls, is the use of long-read sequencing as the long-reads can span repetitive regions and improve mapping to the reference genome. Long-read sequencing technologies perform better for SV calling than short-read methods [56]. Currently, long-read sequencing is more expensive than short-read sequencing and therefore less used. However, this will ultimately change in the future due to continuous reductions in costs per base using PacBio or Oxford Nanopore sequencing technologies [56]. Therefore, in future dnSV studies, long-read sequencing technologies with adequate read depth will aid in dnSV detection, especially those arising in tandem-repeat sequences which are known to be hypermutable [56].

In this study, we only identified deletions, duplications, and inversions. Other SV types such as translocations and insertions, were not detected due to the software we used (Lumpy) and were assigned to the “breakend” class (breakpoints that cannot be assigned to a certain SV type). In our dataset, ~ 34% of SVs were assigned to the “breakend” class. The majority of these SVs are repetitive elements leading to false positive and false negative variant calls [55], but also translocations and insertions were assigned to this class. Thus, dnSV detection is also dependent on the type of SV caller, as several SV callers are designed to detect only certain SV types [55, 56].

Our estimate of the swine germline dnSV rate in the current study (0.108 per generation; one dnSV per nine offspring) is similar to that found in a large human cohort of > 4,000 trios (0.122 per generation based on deletions, duplications and inversions, using similar data, methods and criteria) [6]. Nevertheless, our dnSV estimate is preliminary given the small sample size in our study, and likely conservative due to the detection of dnSVs using short-read sequencing data and that we only focused on detection of deletions, duplications, and inversions. Furthermore, the human dnSV study found that nearly 73% of dnSVs originated from paternal gametes [6]. In the current study, we were not able to test for a paternal effect because of the small number of dnSVs detected. From the detected dnSVs, we were only able to determine the parent of origin for one dnSV. The other three dnSVs are smaller in size (64, 197, 573 bp) and do not contain any informative SNPs to determine their parent of origin. Future studies with larger sample sizes and long-read sequencing data could contribute to confirming a parental bias in the origin of dnSVs and more accurate detection of dnSV rate.

### Three-generational sequence data

All identified germline dnSVs were found in commercial pig line 1. All trios from line 1 had genomic DNA isolated from ear punch and the trios from line 2 had genomic DNA isolated from ear punch, hair, or semen, where some semen samples showed poor sequencing quality. In line 1, some trios had three-generational sequence data. Two of the four dnSVs were detected in probands with one sequenced third-generation offspring available and transmission of both dnSVs was confirmed. Due to poor sequencing quality of some semen samples and lack of three generational sequence data, it was challenging to confidently detect dnSVs in pig line 2. We were able to validate two out of three PCR tested dnSVs in the proband and some of its offspring. The dnSV which was not validated by PCR, the 573 bp duplication, was, however, validated by confirming transmission in the probands’ sequenced offspring. Mutation cluster 2 was not validated as it was too complex for PCR primer design and the proband did not have sequenced offspring available. Additionally, the 276 bp mosaic deletion was not validated by PCR and due to lack of sequenced offspring of the proband, we were not able to derive whether this mosaicism was restricted to the soma or the germline. Thus, three-generational sequence data, including sequenced offspring of the proband will aid in detection and validation of germline dnSVs. We suggest future studies to sequence a larger number of third-generation offspring to increase probabilities of having at least one offspring which inherited the dnSV from the proband [2]. This is more feasible in livestock species compared to humans due to their population structure. In humans, a minimum of four third-generation offspring was shown to be sufficient [6]. However, in livestock, it is feasible to sequence at least eight to ten third-generation offspring, which increases the probability of sequencing at least one offspring that inherited the dnSV from 93.75% (sequencing four third-generation offspring) to 99.6–99.9%. Livestock species, including pigs, provide an opportunity to study dnSVs due to their population structure and routine collecting of data, contributing to a better understanding of genetic disorders and evolutionary patterns.

## Conclusions

The current study focused on identification and characterisation of dnSVs in pigs using trio-based whole genome sequence data. We identified four germline dnSVs, all overlapping with intronic regions of protein-coding genes. Two of the four dnSVs are clustered mutations where a dnSV co-occurs with other dnSVs or with a dnSNV. Our estimate of the swine germline dnSV rate is 0.108, which is a conservative estimate, given our small sample size and the challenges of dnSV detection from short reads. The sample size and the dnSVs that are smaller in size in the current study precluded observation of any paternal bias in the origin of dnSVs. Future dnSV studies will benefit from larger cohorts of trios with three-generational long-read sequence data. The current study highlights the complexity of dnSVs and shows the potential of breeding programs for pigs and livestock species in general, to provide a suitable population structure for identification and characterisation of dnSVs.

## Methods

### Animal samples

The dataset included 117 individuals from 46 sire-dam-proband trios of two commercial pig lines, line 1 (55 samples, constituting 22 trios) and line 2 (62 samples, constituting 24 trios). All trios from line 1 had genomic DNA isolated from ear punch. The trios from line 2 had genomic DNA isolated from ear punch, hair or semen. All trios had two-generation sequence data (WGS of parents and the proband) available. Additionally, some trios of line 1 had three generations of sequence data; four probands were used as sires in other trios, resulting in their offspring having sequence data available. All samples were whole genome sequenced (mean sequencing depth = 32.4X) using Illumina paired-end sequencing, with 150 bp read length and 300 bp fragment length. The paired-end reads were realigned to the pig reference genome (Sscrofa11.1, GenBank assembly accession number GCF_000003025.6) with the Burrows-Wheeler Aligner (BWA-mem v.0.7.17) [59] to generate a BAM file for each individual. All BAM files were sorted and indexed with SAMtools (v.1.9) [60]. Sequence alignment and variant calling was performed on the High Performance Computing (HPC) cluster at Wageningen University and Research. Nine trios (21 samples) were excluded because of poor data quality including large numbers of discordantly mapped reads.

### SV detection

We used a pipeline (v.0.1.0) [61] to perform structural variant (SV) calling in a population using Smoove (v.0.2.8) [62]. This Smoove pipeline used ‘Lumpy’ (v.0.2.14a) to call SVs in each sample relative to the reference genome. Lumpy uses signals from split reads and discordant paired end reads to predict breakpoints of deletions, duplications, and inversions [63]. Four different types of SVs are identified with Lumpy: deletion, duplication, inversion and breakend variants that cannot be assigned to one of these three classes (therefore ignored in current study) [30]. SVtools (v.0.4.0) was used to combine all SV calls into one single variant call format (VCF) file [64]. Subsequently, all 37 trios were genotyped for population-wide non-redundant SV sites with SVTyper (v.0.7.0), which uses a Bayesian framework that uses allele counts at each junction to determine the likelihood that a genotype is heterozygous or homozygous [65]. The VCF file was re-genotyped with SVTyper to get information for all SVs in all samples for filtering. The population SV pipeline generated a VCF file in which all detected SVs were denoted, and each sample was assigned a genotype for each SV.

### ***De novo*** candidate filtering

We filtered SVs using BCFtools (v.1.9) [66], VCFtools (v.4.0.0) [67], and custom R (v.3.6.2) [68] and Python (v.2.7.15) [69] scripts. SVs were declared a dnSV when the proband was heterozygous for the SV and parents and unrelated trios did not have the SV. The sum of genotype quality (GQ) scores for a trio had to be greater than 120. In addition, allele frequencies of dnSVs higher than 0.1 across the whole dataset were excluded, because dnSVs are unique and expected to be a one-time event. We chose this threshold of 0.1, to account for potential dnSV transmissions from proband to sequenced third-generation offspring present in the dataset.To filter out spurious false dnSVs, we included additional filters per SV type. Deletions passed if the median depth inside the dnSV compared to the median depth 1,000 bases left and right of the dnSV (duphold flank fold-change [DHFFC]) [70] was < 0.8; average DHFFC of sire and dam was > 0.8; minimum allelic balance, fraction of reads supporting the alternate allele out of the reads supporting reference and alternate allele, for proband was > 0.05; there were a minimum of three reads supporting the dnSV in the proband; maximum allelic balance for parents was < 0.1; a maximum of three reads supporting the dnSV was present in either parent; and the deletion was not called a dnSV in > 1 sample.

Duplications passed if the median depth inside the dnSV compared to the median depth of bins with matching GC content (duphold bin fold-change [DHBFC]) [70] was > 1.1; average DHBFC of sire and dam was < 1.2; minimum allelic balance for proband was > 0.1; there was a minimum of three reads supporting the dnSV in the proband; maximum allelic balance for parents was < 0.1; a maximum of three reads supporting the dnSV in either parent; and the duplication was not called a dnSV in > 1 sample.

Inversions passed if the minimum allelic balance for the proband was > 0.2; there were a minimum of five reads supporting the dnSV in the proband; maximum allelic balance for parents was < 0.1; there was a maximum of three reads supporting the dnSV in either parent; and the inversion was not called a dnSV in > 1 sample.

Finally, candidate sites were manually inspected using Integrative Genomics Viewer (IGV) (release 2.11.9, December 2021) [31] to remove spurious dnSV cases. Manual inspection included some stringent criteria and dnSV cases were considered spurious when (1) both breakpoints of a dnSV were overlapping with repeats, (2) only one side of the dnSV breakpoint was supported with split or paired-end reads, (3) less than three split or paired-end reads supported the dnSVs at each side of the breakpoint, (4) the split or paired-end reads supporting a dnSV breakpoint were not at all overlapping with each other, or (5) there was no clear visual increase (for duplications) or decrease (for deletions) in coverage at both sides of the dnSV breakpoints.

The dnSV rate was estimated by dividing the number of identified true dnSVs with the number of trios analysed. Confidence intervals of the dnSV rate estimate were calculated using the Wilson method [71].

### Validation of dnSVs

The detected dnSVs were compared with the public SV database of Ensembl (release 107, July 2022) [32] and with a pig-specific variation database, PigVar [33] (accessed at 12 November 2022), to validate that these identified dnSVs were novel variants not reported before in pigs.

PCR primers were designed to span each of the breakpoints identified in the sequence data. Mutation cluster 2 was too complex for PCR primer design. For the remaining three dnSVs and the mosaic deletion, PCR assays were designed (Additional file 2: Table S1). The primers were tested on DNA from the proband, its offspring, and its parents. The PCR was performed on 60ng of DNA (6ul), 0.4 μm primer (0.06ul), 2.5ul FirePol 5x Master Mix and 3.5ul MQ. The PCR cycling conditions were 95 °C for 5 min; 35 cycles of 30 s at 95 °C, 45 s at 55 °C annealing temperature, 90 s at 72 °C; followed by a final elongation step of 72 °C for 10 min. The PCR products were loaded on 3% agarose gel.

Furthermore, two dnSVs, the 64 bp deletion and the 573 bp duplication were detected in probands with three generations of sequence data. Hence, the breakpoints of these dnSVs were visualised in sequenced offspring of the corresponding proband using IGV in order to confirm transmission of these dnSVs.

### Determining the parent of origin

Presence of informative SNPs located within dnSVs aided in identifying parent of origin. For deletions, the informative SNPs we used were homozygous for the alternate or reference allele in the proband, and homozygous for the other allele in one of the parents. The parent of origin could then be determined to be on the haplotype of the parent who is homozygous reference for the informative SNP alleles [30].

For duplications, informative SNPs were heterozygous with a 2:1 ratio and one parent homozygous (for either allele) and the other heterozygous. The allele with a higher than expected number of reads (causing the 2:1 ratio) indicated the parental haplotype the duplication originated from [30].

### Functional annotation

Genes located within the dnSVs were retrieved from Ensembl (release 107, July 2022) and NCBI annotation (release 106, May 2017) using NCBI Genome Data Viewer [72]. GeneCards [73] was used to gain insight in the functional enrichment of genes. The Mouse Genome Database [41] was used to look for phenotypic effects in knockout mice.

### Prediction of causal mechanisms

We identified the most likely causal mechanisms for dnSV formation based on split reads spanning the breakpoints of the dnSVs. The dnSVs were grouped into two broad categories based on the degree of sequence homology at the junctions, using methodology from a similar analysis of dnSVs in human families [6]. dnSVs which showed no breakpoint homology (0 to 1 bp) were grouped as the non-homology based category (NON-HOM), of which non-homologous-end-joining is most often the cause of this type of mechanism [6]. Additionally, dnSVs which showed 2 to 15 bp sequence homology flanking the breakpoints were grouped as the micro-homology based category (MICRO-HOM) [74]. Two main mechanisms of this class are micro-homology-mediated break-induced replication and microhomology-mediated end joining [52, 74, 75].

## Electronic supplementary material

Below is the link to the electronic supplementary material.

Supplementary Material 1

Supplementary Material 2

## Data Availability

The data that support the findings of this study are available from Hendrix Genetics B.V. and Topigs Norsvin but restrictions apply to the availability of these data, which were used under license for the current study, and so are not publicly available. Data are however available from the authors upon reasonable request and with permission of Hendrix Genetics B.V. and Topigs Norsvin.

## List of abbreviations

DNM: *de novo* mutation
dnCNV: *de novo* copy number variant
dnSNV: *de novo* single nucleotide variant
dnSV: *de novo* structural variant
MICRO-HOM: micro-homology
NON-HOM: non-homology
SNP: single nucleotide polymorphism
VCF: variant call format
WGS: whole genome sequencing
