# Supplementary material for: Identification and characterisation of de novo germline structural variants in two commercial pig lines using trio-based whole genome sequencing
Source: BMC Genomics. 2023 Apr 18;24:208. doi: 10.1186/s12864-023-09296-3 (PMC10114323; doi:10.1186/s12864-023-09296-3)
Supplement: Supplementary file 2 — Supplementary Material 2 [file 12864_2023_9296_MOESM2_ESM.docx]

**Figure S1. The number of variants per sample plotted against the mean sequencing depth per sample for each variant type**


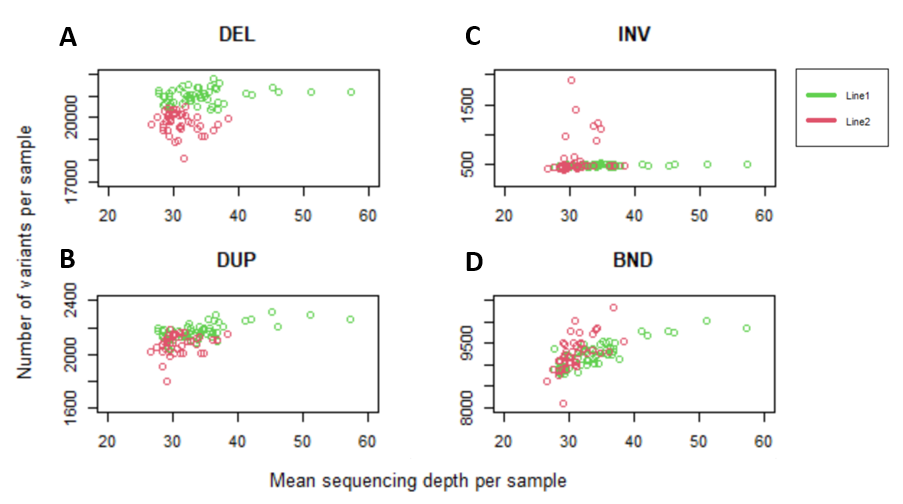


Samples of the 37 analysed trios were included (55 samples from commercial pig line 1 and 41 samples from commercial pig line 2). **A.** Number of deletions per sample plotted against mean sequencing depth. **B.** Number of duplication per sample plotted against mean sequencing depth. **C.** Number of inversions per sample plotted against mean sequencing depth. **D.** Number of ‘breakend’ class plotted against mean sequencing depth.

**Figure S2. IGV screenshot of mutation cluster 1**


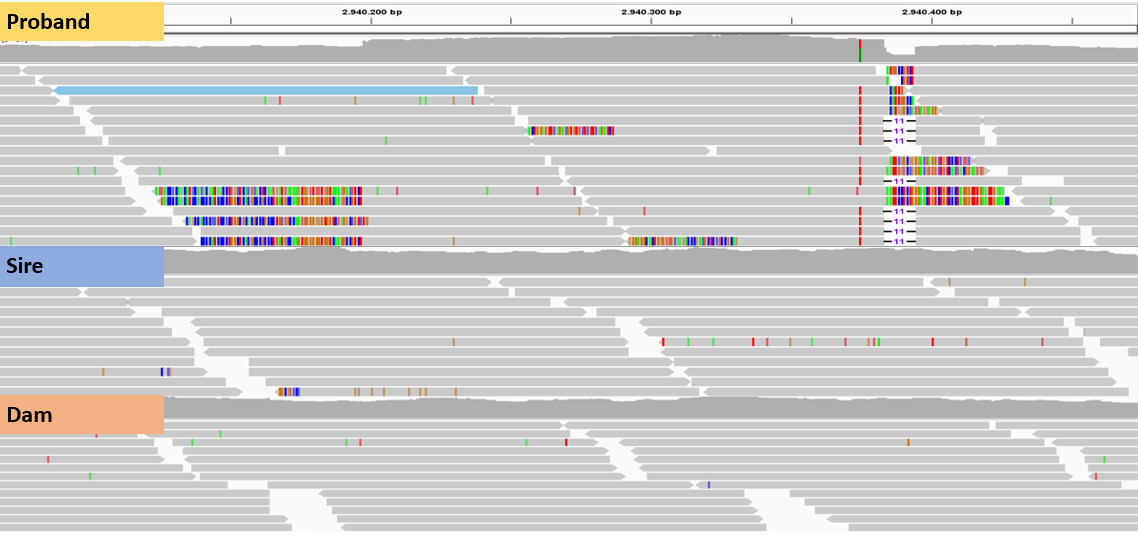


Colorful reads indicated split reads which supported the 187 bp *de novo* duplication chr1:2940197-2940384 in the proband. Red vertical stripes indicated a *de novo* single nucleotide variant (dnSNV) on chr1:2940375 in the proband and the 11 bp stripes indicate a 11bp *de novo* deletion (indel) on chr1:2940383-2940394 in the proband.

**Figure S3. IGV screenshots of mutation cluster 2**


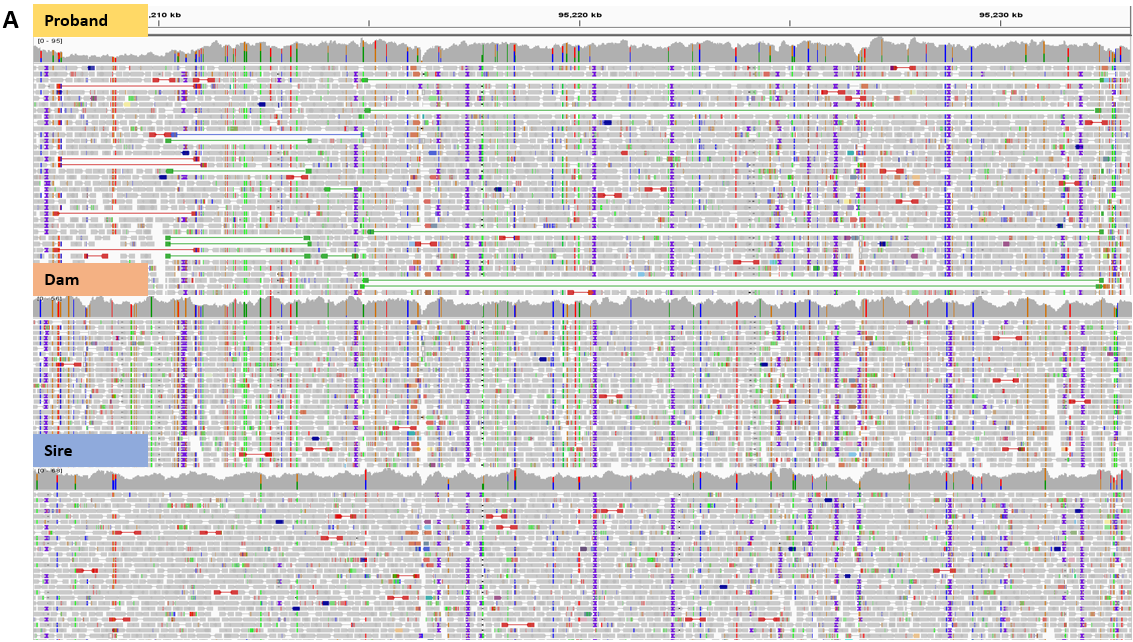


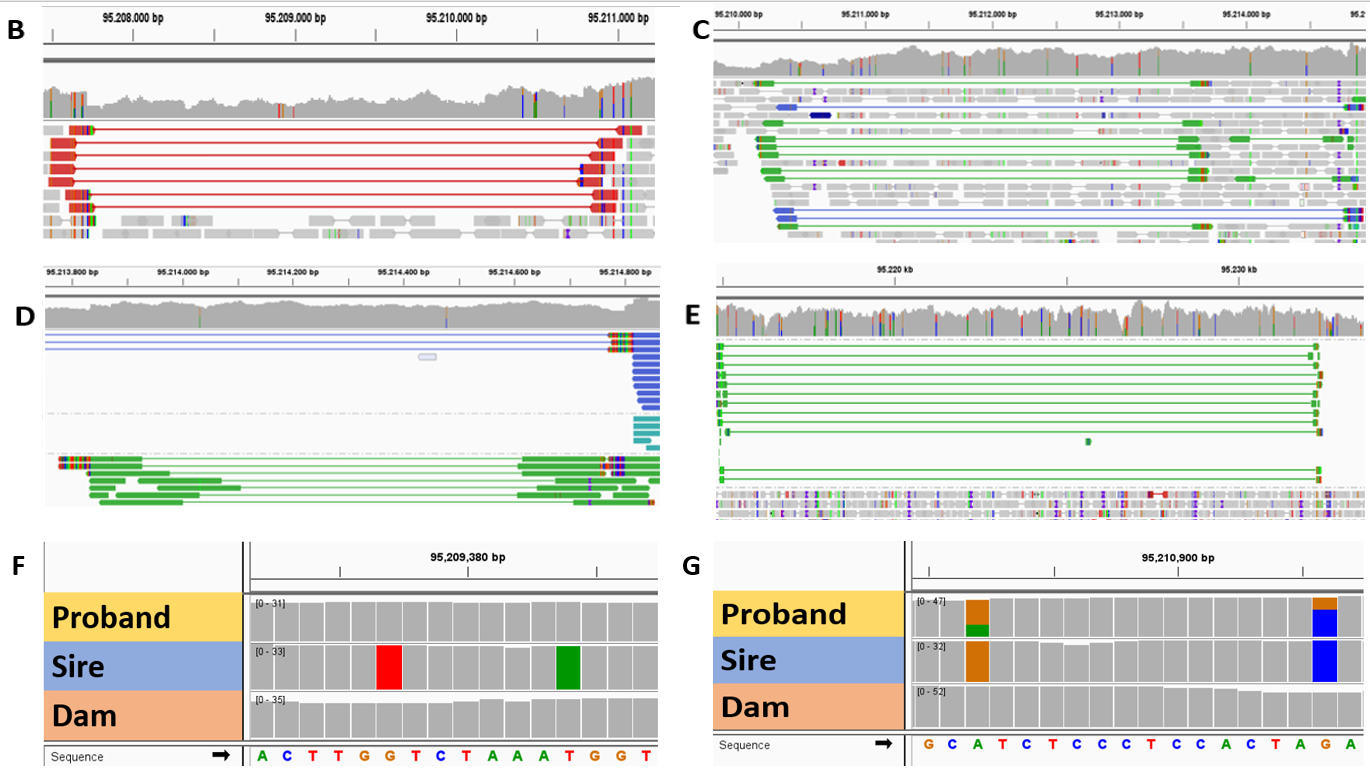


**A.** IGV screenshot of mutation cluster 2 including one *de novo* deletion and three *de novo* duplications, of which one was inverted. **B.** Red lines indicated the 3,072 bp deletion on Chr1:95207720-95210792. **C.** Green lines indicated the 3,456 bp inverted duplication on Chr1:95210182-95213638. **D.** Green lines indicated the 1,008 bp duplication on Chr1:95213834-95214842. **E.** Green lines indicated the 17,660 bp duplication on Chr1:95214800-95232460. **F.** An example of two informative SNPs within the 3,072 bp deletion: SNPs are homozygous for the reference allele in the proband and homozygous for the alternate allele in the father. **G.** An example of two informative SNPs within the 3,456 bp inverted duplication: SNPs are heterozygous with a 2:1 ratio in the proband where the allele with more than expected number of reads (causing the 2:1 ratio) came from the father.

**Figure S4. IGV screenshot of the 573 bp *de novo* duplication**

**
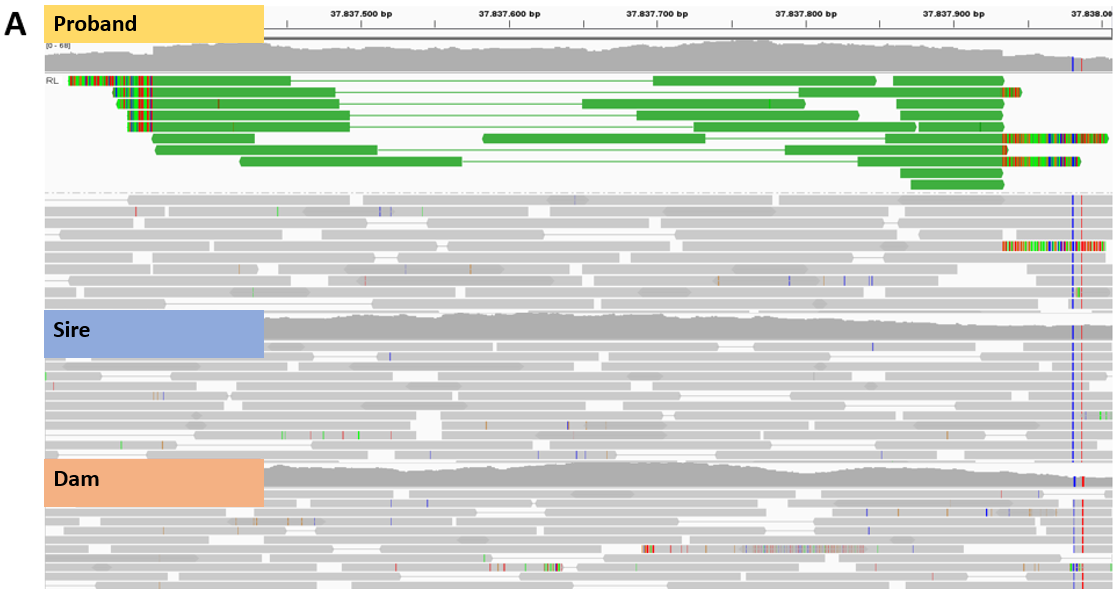

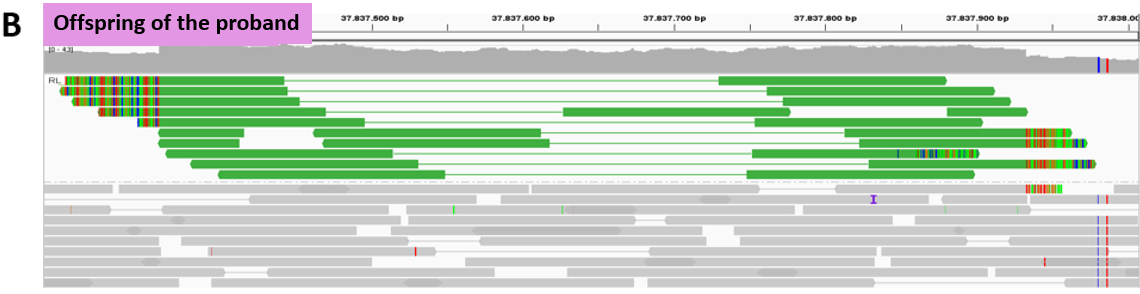
**

573 bp *de novo* duplication found on chr7:37837360-37837933. **A.** Green lines indicated the *de novo* duplication in the proband and absence of green lines in parents indicated absence of the duplication in both parents. **B.** The *de novo* duplication transmission confirmed in one sequenced offspring of the proband.

**Figure S5. IGV screenshot of the 64 bp *de novo* deletion**

**
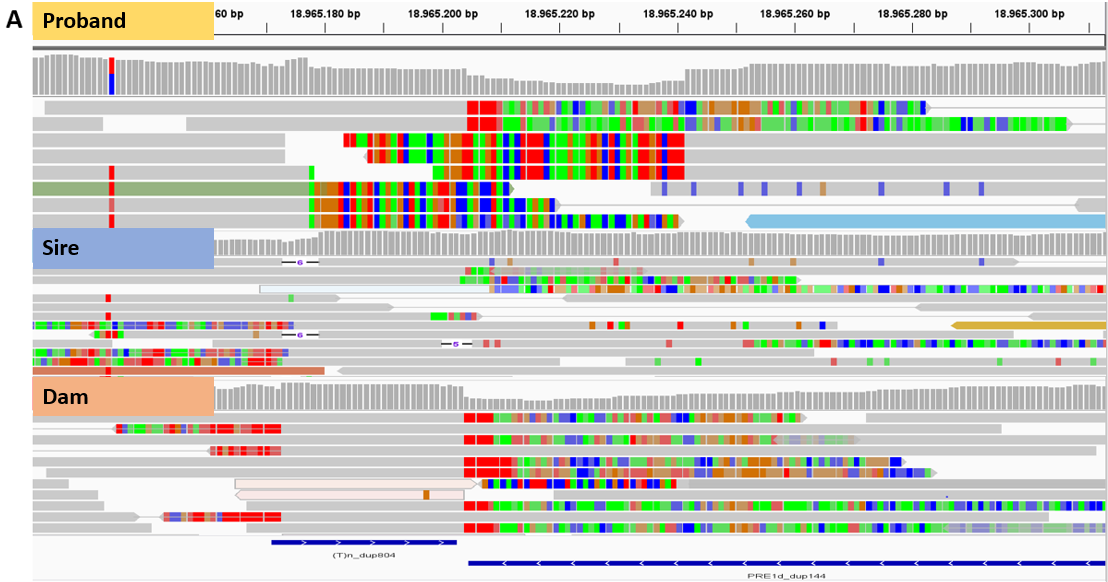
**

**
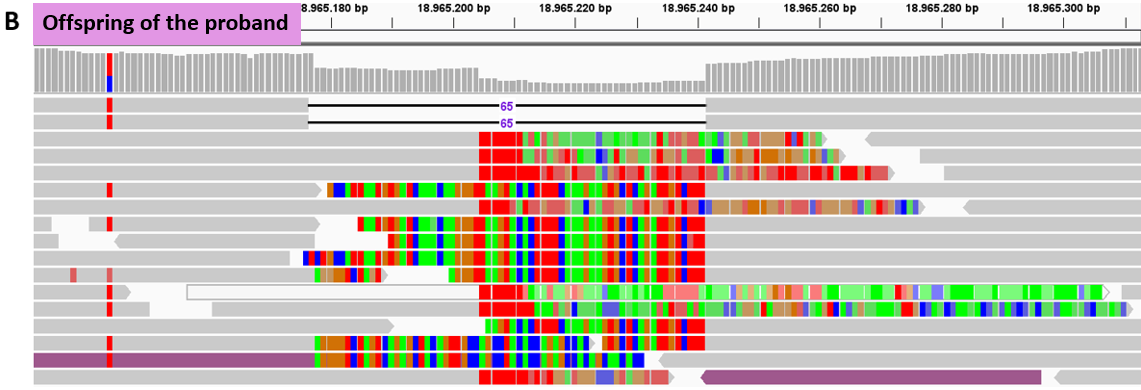
**

64 bp *de novo* deletion found on chr15:18965177-18965241. **A.** Colorful reads indicated split reads supporting the *de novo* deletion in the proband and absence of the same colorful reads in parents supported evidence for absence of the deletion in the parents. Repeatmasker showed that repeats overlapped with this region. **B.** The *de novo* deletion transmission was confirmed in one sequenced offspring of the proband.

**Figure S6. IGV screenshot of the 276 bp mosaic deletion**


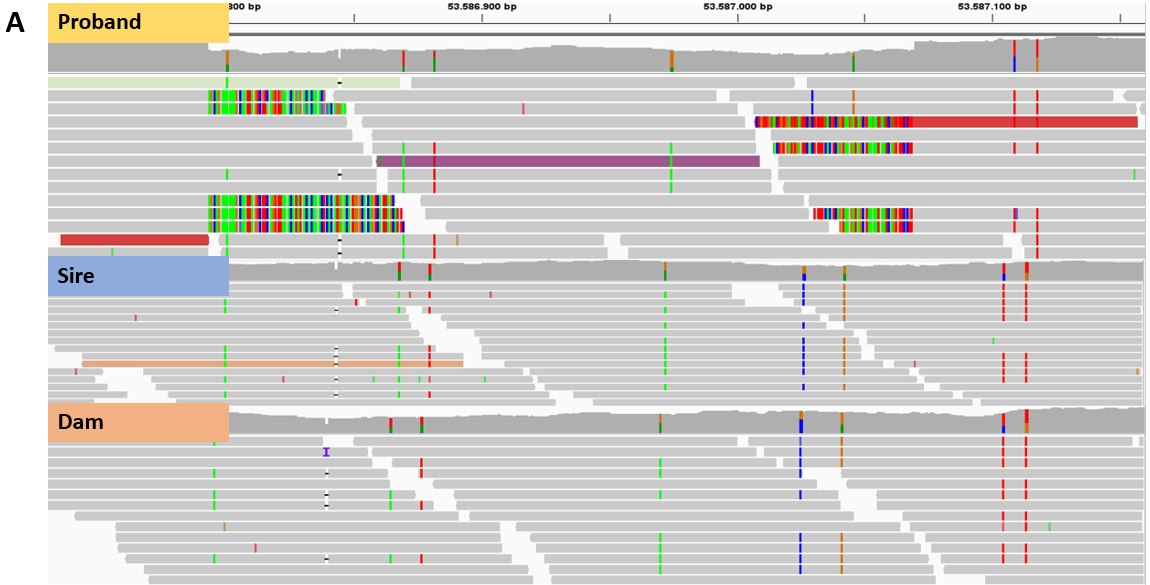

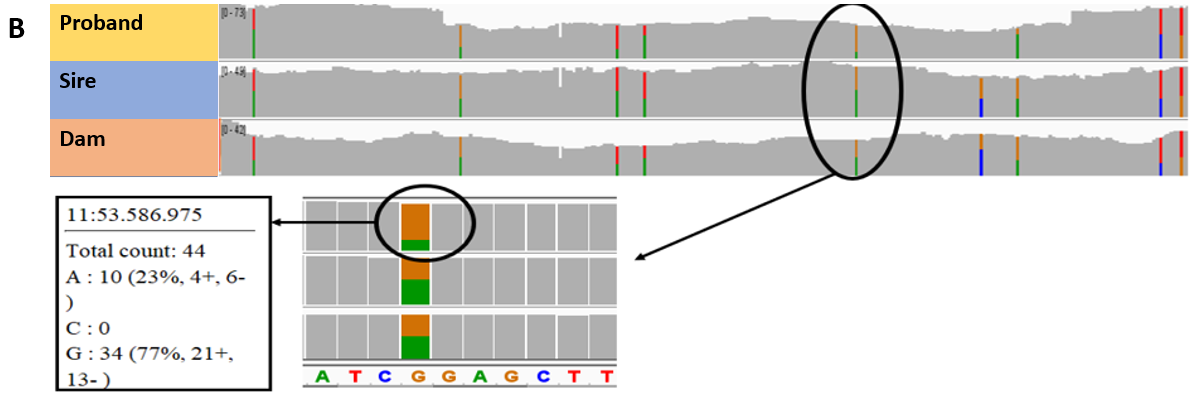
 276 bp mosaic deletion found on chr11:53586793-53587069. **A.** Screenshot representing the reads supporting evidence for the mosaic deletion. Red and colorful reads supported evidence for the deletion in the proband. Absence of these red and colorful reads in parents indicated no deletion. **B.** Screenshot of the sequence coverage within this deletion. Heterozygous single nucleotide polymorphisms (SNPs) within the deletion showed 2:1 ratio in proband and 1:1 ration in both parents. The SNP count indicated that 25% is deleted. This deviation of the 50% deletion expected true germline *de novo* structural variants provided evidence for a mosaic deletion.
